# Disorders of compulsivity: Deficits in arbitrating learning strategies

**DOI:** 10.1111/adb.13433

**Published:** 2024-08-09

**Authors:** Zhongqiang Ruan, Shilin Liu, Yu an Liu, Qiong Yang, Ziwen Peng

**Affiliations:** ^1^ School of Psychology, Center for Studies of Psychological Application, and Guangdong Key Laboratory of Mental Health and Cognitive Science South China Normal University Guangzhou China; ^2^ Key Laboratory of Brain, Cognition and Education Sciences, Ministry of Education Guangzhou China; ^3^ The Affiliated Brain Hospital, Guangzhou Medical University Guangzhou China

**Keywords:** arbitration control, compulsivity, model‐based, model‐free

## Abstract

While previous research has shown that compulsivity is related to an imbalance between goal‐directed and habitual learning systems, very little is known about whether this effect is due to the impairment of a single system or the impairment of the arbitration mechanism that determines which system controls behaviour at any given moment; the current study aims to address this disagreement. Nineteen alcohol use disorder, 30 obsessive‐compulsive disorder (OCD) and 20 major depressive disorder patients and corresponding sex‐ and age‐matched controls performed two‐choice, three‐stage Markov decision‐making paradigm. Model‐based and mode‐free reinforcement learning models were used to independently fitted their behavioural data. Alcohol use disorder and OCD patients showed less model‐based strategy choice than healthy controls in task conditions where the model‐based strategy was optimal. Only OCD patients showed higher behavioural control system switching in task conditions where model‐free use was optimal. Major depressive disorder patients did not differ from the matched control in both. These findings suggest that dysfunction in arbitration control between dual systems may be the basis for diverse disorders involving compulsivity.

## Graphical abstract

Under complex conditions where implementing model‐based strategies is optimal, the ability of alcohol use disorder and obsessive‐compulsive disorder (OCD) patients to inhibit shifting to a model‐free system of responding is impaired. Major depressive disorder patients without OCD comorbidities showed no difference in arbitration control compared to matched healthy controls. This study suggests that abnormal arbitration control may be an underlying mechanism leading to mental disorders characterized by compulsive behavior.

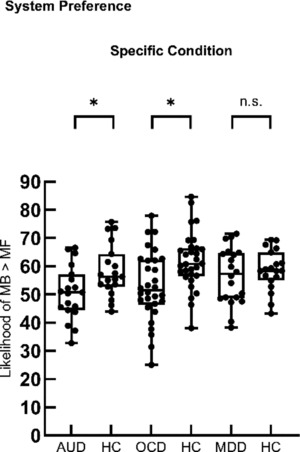

## 1 INTRODUCTION

In the pathological behaviour of disorders, such as alcohol use disorder (AUD), one of the puzzling characteristics is that individuals will repeatedly choose to perform behaviours despite knowing that performance of such behaviours will produce strong negative consequences. Persistent behaviour, which is inappropriate for the situation, has no obvious relationship to the overall goal and often leads to adverse consequences, is defined as compulsivity.^1^ One explanation for compulsive behaviour^2^ is that decisions can arise from two different, parallel instrumental control systems, known as goal‐directed and habitual control.^3^, ^4^ In goal‐directed control, the possible outcomes predicted by environmental models guide us in making choices. In habitual control, previous rewards reinforce our repeated choices. Findings in human studies suggest that compulsivity is associated with weakened goal‐directed learning and potential increases in habitual learning.^5^, ^6^ However, the mechanism behind these behavioural changes remains unclear. Research suggests these changes may be the result of one or a combination of several of the following mechanisms: a separate increase in the intensity of habitual control, a separate decrease in the intensity of goal‐directed control or a separate damage in the arbitration mechanism for deciding which control system to use for a given choice.^7^ The current study utilized a three‐stage sequential learning task and computational modelling to test the third hypothesis, which is that disorders of compulsivity are related to an impaired arbitration mechanism, resulting in deficits in choosing the optimal system to control behaviour.

Computational neuroscientists have investigated how compulsivity is associated with imbalanced goal‐directed and habitual control using a two‐step sequential learning task developed from a reinforcement learning framework.^8^, ^9^ Specifically, in the two‐step task, goal‐directed behaviour is assessed by model‐based (MB) reinforcement learning, which examines all possible combinations of actions and outcomes based on a decision tree, uses state prediction error (SPE) signals to compute and update action value and is sensitive to changes in the task structure. Conversely, habitual behaviour is assessed by model‐free (MF) reinforcement learning, which computes and updates action value using reward prediction error (RPE) signals based on previous stimulus response. Among these, the former is forward‐looking and more flexible, but computational expense is higher, whereas the latter is retrospective and inflexible, but computational efficiency is higher. Recently, researchers put forward the hypothesis of dynamic arbitration control, purporting that there is an independent arbitration mechanism that decides the weights of MB and MF systems according to the requirements of the task environment to achieve the optimal selection of behaviour control strategies.^10^, ^11^, ^12^ Here, the estimation of the prediction uncertainty of the controller mediates a trade‐off between the two; when the prediction of MF control is more accurate, it has a greater weight, but when the prediction of MF control becomes unreliable, MB control is assigned a greater weight.

Recent research has found that both obsessive‐compulsive disorder (OCD) and high Obsessive‐Compulsive Inventory‐Revised (OCI‐R)^13^ scores are related to impaired arbitration between MB and MF reinforcement learning. Patients found it difficult to increase the use of the goal‐directed system in complex environments and maintain the use of the habitual system in simple environments, the latter also occurring in subclinical populations.^14^ Compulsivity is the core symptom of OCD; however, this fact alone does not help us determine whether disorders of compulsivity are related to impairment of the arbitration mechanism between the two systems. Compulsive behaviour is also a characteristic of AUD,^15^, ^16^, ^17^ and AUD is associated with imbalance in psychological structures related to goal‐directed and habitual decision‐making. For example, AUD patients showed evidence of less use of goal‐directed choices, but no difference was observed in habitual choice between these individuals and healthy controls (HCs).^18^ A functional magnetic resonance imaging study reported that the engagement of regions implicated in goal‐directed control (e.g., the ventromedial prefrontal cortex and anterior putamen) in AUD patients decreased, while the engagement of regions implicated in habitual control (e.g., posterior putamen) increased.^19^ A longitudinal tracking study found that MB control was negatively correlated with binge drinking behaviour, while MF RPE signals in the ventromedial prefrontal cortex and ventral striatum were associated with alcohol consumption score development.^20^ Deficits in increasing the use of goal‐directed control can lead to individuals being divorced from their goals, which means that an impaired arbitration mechanism may underlie compulsivity in both OCD and AUD. In summary, existing research has yet to clarify whether the imbalance between two systems related to compulsivity is due to a single system anomaly or arbitration damage.

To further examine this question, the current study employed a three‐stage reinforcement learning task that was used previously to show that healthy volunteers chose and switched different learning strategies according to the context.^10^ The trial‐by‐trial computational modelling of subject behaviour data can separate goal‐directed, habitual and arbitration processes, independently evaluating the contribution of each system. In this study, we examined the performance of two groups of subjects diagnosed with AUD and OCD, as well as corresponding sex‐ and age‐matched control subjects. Additionally, in order to rule out the impact of comorbidities of depression, the performance of subjects diagnosed with major depressive disorder (MDD) and matched control subjects was also compared. We predicted that disorders of compulsivity would be related to impaired arbitration between MB and MF learning.

## 2 METHODS

### 2.1 Subjects

AUD patients were recruited from inpatients at the Affiliated Brain Hospital of Guangzhou Medical University, while OCD and MDD patients were recruited by clinicians from an outpatient population. Among these, the data of OCD patients have been previously reported.^14^ Three HC groups matched in sex and age with each of the three patient groups were recruited via community and university‐based advertisements in the Guangzhou region. Diagnoses were confirmed by psychiatrists using structured clinical interviews (i.e., the MINI‐International Neuropsychiatric Interview^21^) and criteria for AUDs, OCD or MDD found in the Diagnostic and Statistical Manual of Mental Disorders, Version V. In our sample, three of the AUD patients had a comorbid depression, none of which had comorbid OCD; nine of the OCD patients also exhibited comorbidity: three with depression, six with anxiety and three with anxiety and depression; none had comorbid AUD. Only MDD patients without comorbidity of AUD and OCD and with an OCI‐R scores less than 21 were included. All patients were undergoing treatment with medication (more details are described in Table S1). HCs did not use psychotropic drugs or experience medical, neurological or mental illnesses. After the experiment, subjects received a bonus of at least 30 RMB based on their task performance.

### 2.2 Clinical assessments

AUD patients and matched controls completed the Alcohol Use Disorders Identification Test to assess the severity of AUD symptoms.^22^ OCD patients and matched controls completed the Yale‐Brown Obsessive‐Compulsive Scale to assess the severity of OCD symptoms.^23^ All subjects completed the OCI‐R to characterize categories of OCD symptoms^13^ and the Beck Depression Inventory to assess depressive symptoms.^24^ Furthermore, we used the State–Trait Anxiety Inventory to estimate anxiety symptoms^25^ and the Barratt Impulsiveness Scale‐11 to estimate impulsiveness behaviours.^26^

### 2.3 Task

**FIGURE 1:**
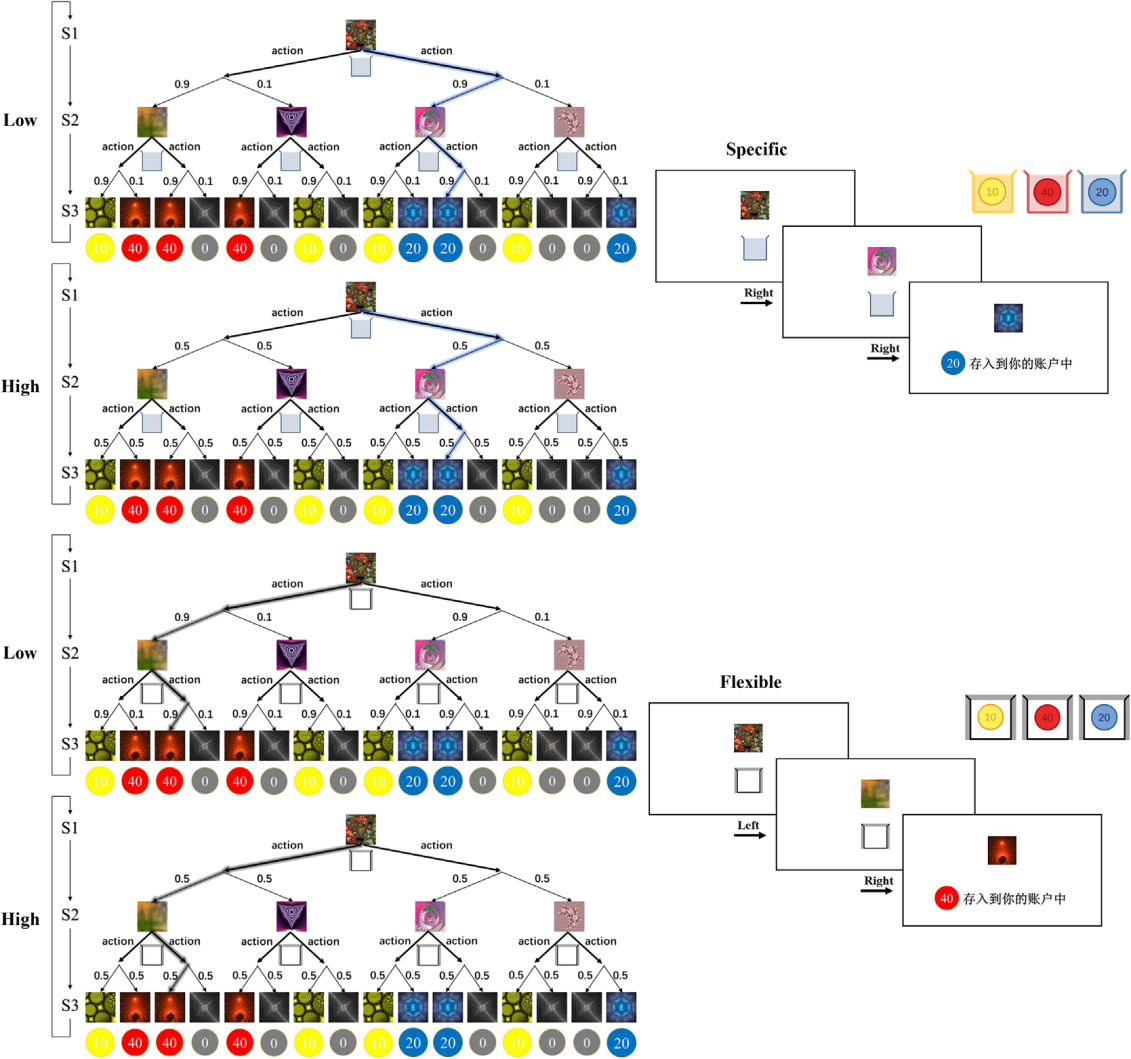
Task structure and example of optimal choices. The fractal image represents the state of the agent. The colour of the collection box indicates the target assigned in each trial. The thick arrow represents the agent's choice, and the thin arrow corresponds to the state transition probability. Subjects make two consecutive choices based on each task target to obtain a reward in each final stage.

Subjects received extensive guidance from the experimenter and were provided with practical examples of the structure of a sequential two‐choice Markov decision task.^10^, ^27^ The content of the guidance was based on the subjects' understanding and generally lasted for 10–20 min. As shown in Figure 1, each participant started from the same state (S1) each time, pressing the left or right button to make a decision, transitioning to the second stage state (S2) through probability state transition, then executing the second of two decisions and reaching a final state (S3) associated with coins (0–40) through the same probability state transition. Although different subjects could have randomly assigned decision trees with the same task structure but different coin colours and fractal image locations, once the experiment began, the decision tree would remain unchanged, giving subjects the opportunity to explore and learn the possible outcomes of probabilistic state transitions.

The experimental paradigm was divided into two different task conditions: the specific goal condition and the flexible goal condition. The specific condition was set to increase the use of MB strategy by subjects, so only coins of specific colour (yellow, red or blue) would be accepted each trial. In this case, the subjects had to rely on establishing an internal understanding of the task structure to obtain coins. On the contrary, the flexible condition was designed to promote subjects to adopt MF strategy, so all coloured coins could bring rewards. At this point, an MB strategy in early experience would give way to an MF strategy after more experience; subjects only needed to rely on successful experiences that have been reinforced in the past, regardless of changes in the task environment. At the beginning of each trial, a coloured collection box appeared below the fractal image, indicating whether the goal for the trial is specific or flexible (yellow, red or blue represent specific trial, while white represents flexible trial). There was a time limit of 2 s for each choice, and the next state will appear 150 ms after making the decision. And once the subject reached the third and last state of each trial, the collection box would disappear and be replaced by a coin acquisition cue that lasts for 2 s. The coin cues included 10, 20 and 40, each accompanied by one of the three colours of yellow, red and blue. In addition, grey coins were always combined with 0, indicating that the trial did not receive a reward.

To better dissociate MB from MF control and to prevent subjects from using multiple MF strategies in the absence of MB control in specific condition, both conditions included two types of state transition probabilities: high state transition uncertainty and low state transition uncertainty. State transition uncertainty refers to the probability that the choice of the previous stage results in the state entered in the next stage. Decisions made in low uncertainty blocks have a 90% probability of reaching the more likely state, whereas in the high uncertainty blocks, the transition was random (50% probability of reaching each of the next state options; neither of which are indicated to subjects; higher uncertainty increases MF control, while lower uncertainty promotes MB control). Blocks with low state transition uncertainty consisted of three to five consecutive trials, whereas blocks with high state transition uncertainty consisted of five to seven consecutive trials. This formed four different types of blocks (flexible or specific goals combined with low or high uncertainty). The experiment consisted of 14 blocks of each type, which were randomly ordered, that is, an average total of 280 trials. In order to familiarize the subjects with these two conditions, a training phase consisting of 80 flexible goal trials (white collection boxes) and 20 specific goal trials (three colour collection boxes, randomly selected for each experiment) was set before the formal experiment began. The subjects were informed that the task structure during the training phase is the same as the main experimental phase, but the scores during this phase are not included in the total score. The subjects were instructed to collect as many coins as possible, and the total number of coins was related to the actual reward they ultimately received.

### 2.4 Computational model

Consistent with past research,^27^ fitting each subject's choice data using MB and MF reinforcement learning models independently allowed us to quantify the learning preference for MB or MF by estimating and comparing the likelihood of each behavioural control system used in each choice (system preference). In addition, by calculating the frequency of changes between the controller with a higher likelihood in one choice and the controller with a higher likelihood in a subsequent choice, we could quantify the frequency at which subjects switch between the two behavioural control systems (system switching).

In the MF state–action–reward–state–action learner,^28^ the observed reward drove the learning process, and RPEs were used to compute state–action values. Here, $\delta_{\mathrm{RPE}}$ refers to the amount of updates on the state–action value $Q_{\mathrm{MF}}\left(s,a\right)$ of action a in state s:

$$\delta_{\mathrm{RPE}}=r\left(s^{\prime }\right)+\gamma Q_{\mathrm{MF}}\left(s^{\prime },a^{\prime }\right)-Q_{\mathrm{MF}}\left(s,a\right),{\mathrm{\Delta Q}}_{\mathrm{MF}}\left(s,a\right)=\alpha \delta_{\mathrm{RPE}}.$$

s and a are the current state and action, respectively; s′ and a′ are the next state and action in next state, respectively; r(s′) is the reward obtained in state s′; γ is the time discount factor^29^ fixed at 1; and α is the learning rate.

In MB learners,^10^, ^27^, ^29^ the environmental model that represents the probabilities of state–action–state transition was constantly modified by the learning process, and SPEs were used to compute state–action values. The combination of FORWARD learning and BACKWARD planning functions performs state–action value updates.^10^ In the FORWARD learning component, the agent's experience with state transitions was used to compute SPE and update corresponding state–action values:

$$\delta_{\mathrm{SPE}}=1-T\left(s,a,s^{\prime }\right),∆T\left(s,a,s^{\prime }\right)=\eta \delta_{\mathrm{SPE}},Q_{\mathrm{MB}}\left(s,a\right)=\Sigma_{s^{\prime }}T\left(s,a,s^{\prime }\right)\left\{r\left(s^{\prime }\right)+\max_{a^{\prime }}Q_{\mathrm{MB}}\left(s^{\prime },a^{\prime }\right)\right\}.$$

$T\left(s,a,s^{\prime }\right)$ is the matrix of probability of the agent's state being s′ if an action was taken in state s; η is the learning rate; and the first term of the SPE is set to 1: the assumption that the state space is deterministic.

Once the agent has an explicit goal (transition from the specific or flexible to the specific goal condition), it will go through the BACKWARD planning process, which involves repeating the FORWARD update process backward for all possible states and actions in order to update the value of each state.

$$r\left(s\right)=\left\{\begin{array}{c} R\;\text{for}\;a\;\text{goal state},\\ 0\;\text{otherwise}, \end{array}\right.$$

for i = 3, 2,

for $s\in S_{i-1}$,

$$Q_{\mathrm{MB}}\left(s,a\right)=\sum_{s\prime }T\left(s,a,s^{\prime }\right)\left\{r\left(S_{i}\right)+\max_{a\prime }Q_{\mathrm{MB}}\left(s^{\prime },a^{\prime }\right)\right\},\;\text{for}\;\mathrm{all}\;a,$$

end

end

R is the reward value in goal state; $S_{i}$ is the state set of i‐th stage.

The model stochastically selects actions based on the softmax function^30^ below:

$$P\left(s,a\right)=\frac{\exp\left(\mathrm{\tau Q}\left(s,a\right)\right)}{\sum_{b}\exp\left(\mathrm{rQ}\left(s,b\right)\right)}.$$

τ is the inverse temperature parameter, which controls the extent to which the agent chose higher value actions.

Following the same procedure as previously studied,^10^ we use the Nelder–Mead Simplex algorithm^28^ to estimate free parameters of MB and MF learners (learning rate and inverse temperature of the softmax function). The method is to minimize the negative log‐likelihood $-\sum \log\left(P\left(s,a\right)\right)$ of the obtained choice given the observed choices and rewards, summed over all choices for each subject. Optimization was run 200 times using randomly generated seed parameters in order to minimize the risk of finding a local, but not a global optimum. The goodness‐of‐fit measurement estimated using the sum of the negative log‐likelihood across trials is shown in Figure S1.

## 3 RESULTS

In total, 19 AUD patients, 30 OCD patients and 20 MDD patients were compared with sex‐ and age‐matched HCs. The characteristics of the subjects are reported in Table 1.

**TABLE 1:** Demographic and clinical variables.

	AUD (*n* = 19)	HC (*n* = 19)	Statistics		OCD (*n* = 30)	HC (*n* = 30)	Statistics		MDD (*n* = 20)	HC (*n* = 19)	Statistics	
			$\chi^{2}$/t	*p*			$\chi^{2}$/t	*p*			$\chi^{2}$/t	*p*
Sex (M/F)	16/3	11/8	3.199	0.074	16/14	11/19	1.684	0.194	7/13	9/10	0.616	0.433
Age (years)	33.32 (6.01)	30.37 (7.69)	1.316	0.196	24.77 (6.87)	23.00 (1.58)	1.373	0.179	21.25 (3.46)	21.47 (2.09)	0.243	0.810
AUDIT	23.84 (7.72)	0.58 (0.69)	13.084***	<0.001								
Y‐BOCS					23.13 (4.96)	5.97 (4.79)	13.638***	<0.001				
OCI‐R	18.05 (13.34)	12.95 (6.26)	1.510	0.143	27.33 (12.31)	11.37 (4.05)	6.752***	<0.001	13.70 (4.85)	13.32 (2.60)	0.310	0.758
BDI	10.32 (10.06)	8.11 (6.62)	0.800	0.430	20.37 (10.65)	6.00 (5.12)	6.661***	<0.001	20.25 (14.57)	4.95 (4.24)	4.500***	<0.001
STAI‐S	34.68 (10.49)	35.37 (9.74)	0.208	0.836	55.47 (11.03)	35.27 (7.67)	8.236***	<0.001	53.20 (15.56)	32.21 (8.64)	5.243***	<0.001
STAI‐T	41.68 (10.23)	42.53 (8.65)	0.274	0.786	59.00 (8.95)	40.73 (6.82)	8.892***	<0.001	54.40 (12.73)	36.58 (8.47)	5.119***	<0.001
STAI total	76.37 (20.10)	77.89 (16.60)	0.255	0.800	114.47 (18.54)	76.00 (13.75)	9.128***	<0.001	107.60 (28.12)	68.79 (16.50)	5.220***	<0.001
BIS	37.85 (12.72)	34.61 (12.77)	0.785	0.438	49.81 (12.21)	34.44 (9.17)	5.509***	<0.001	48.96 (14.39)	31.89 (9.70)	4.320***	<0.001

Abbreviations: AUD, alcohol use disorder; AUDIT, Alcohol Use Disorders Identification Test; BDI, Beck Depression Inventory; BIS, Barratt Impulsiveness Scale; HC, healthy control; MDD, major depressive disorder; OCD, obsessive‐compulsive disorder; OCI‐R, Obsessive‐Compulsive Inventory‐Revised; STAI‐S, State component of State–Trait Anxiety Inventory; STAI‐T, Trait component of State–Trait Anxiety Inventory; STAI total, Total score of State–Trait Anxiety Inventory; Y‐BOCS, Yale‐Brown Obsessive‐Compulsive Scale.

*** *p* < 0.001.

**FIGURE 2:**
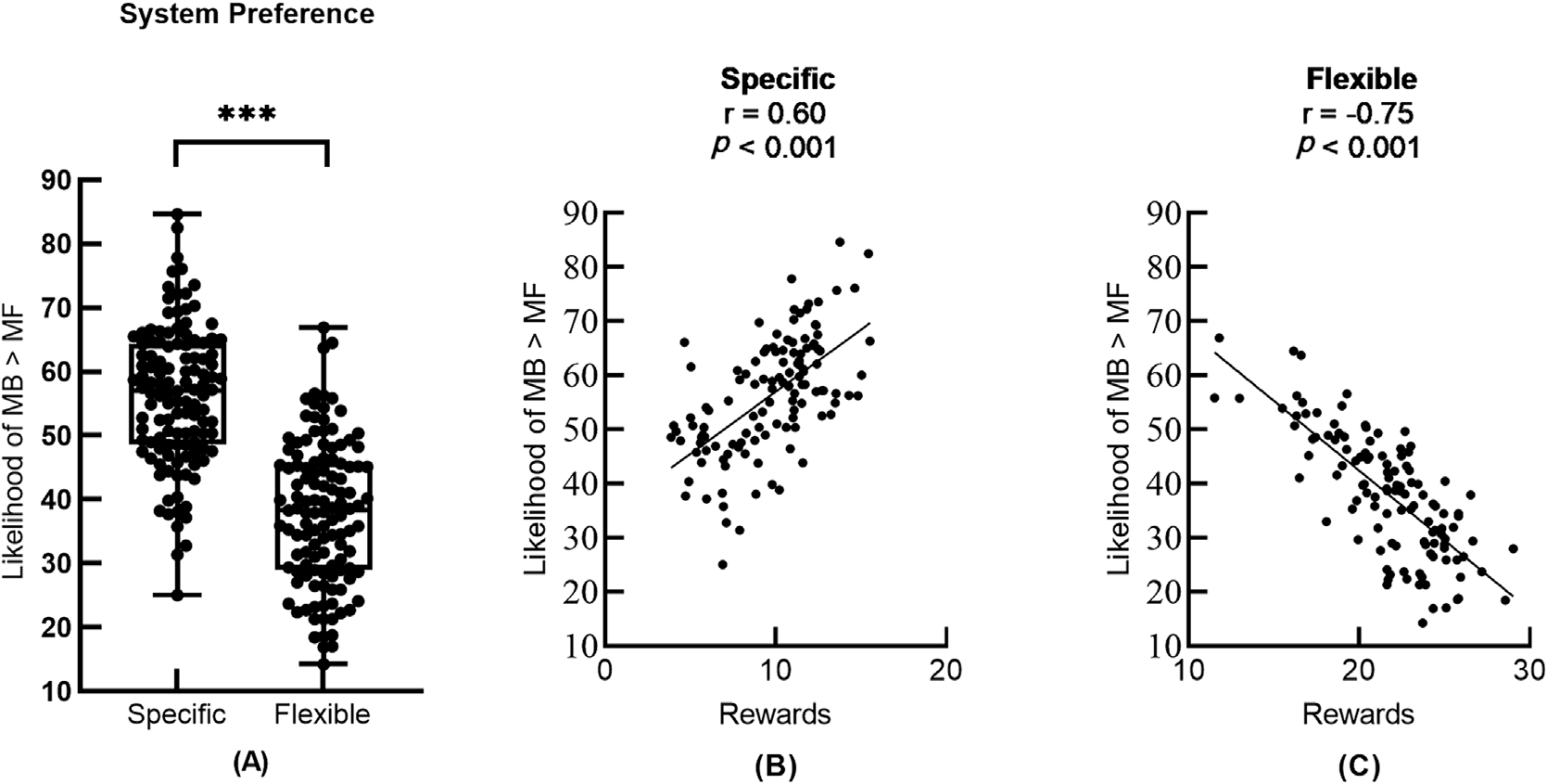
(A) Preference for model‐based (MB) learner (the percentage of choices where an MB controller exhibited higher likelihood than a model‐free [MF] controller) between different task conditions in all subjects. The subjects mainly used MB control in the specific task condition and used MF learning more frequently in the flexible task condition. The preference for MB control was positively correlated with task performance (average number of coins obtained per trial) in specific trials (B) but negatively correlated in flexible trials (C), indicating that the two task conditions are indeed beneficial for the use of different learning strategies. ***p < 0.001.

We analysed the data using a computational learning model, in which the primary outcome was to use multivariate tests to compare model parameters (system preference and system switching) fitted from each individual's trial‐by‐trial choices between each patient group and their own HC group. Consistent with past research,^14^, ^27^ using an MB strategy was better in the specific goal condition, while an MF strategy was optimal in the flexible goal condition (Figure 2).

**FIGURE 3:**
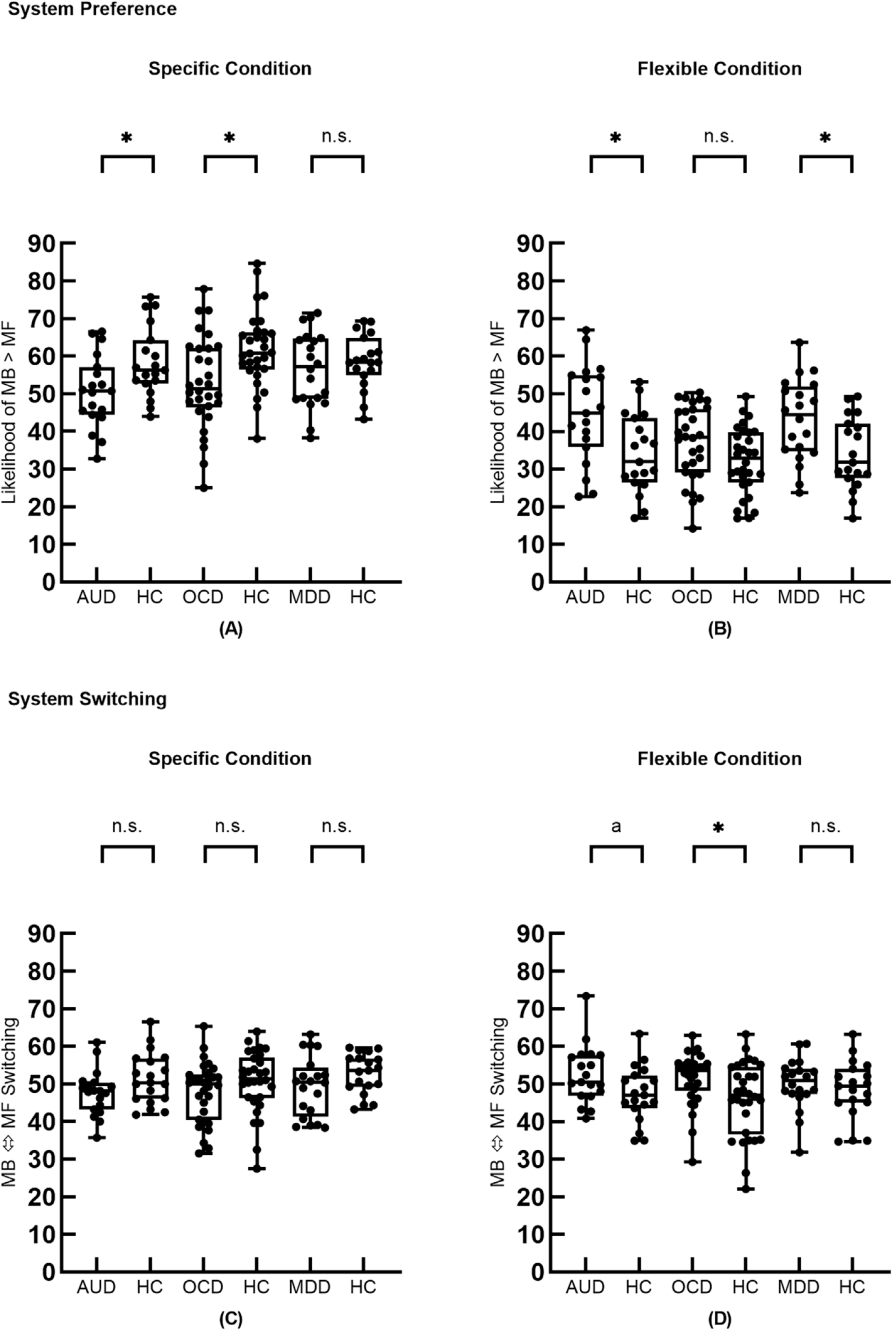
Inferred parameters. Differences in system preferences and system switching (i.e., the percentage of dominant system changes between model‐based [MB] and model‐free [MF] in total system changes) between patients with different disorders and matched healthy controls. AUD, alcohol use disorder; HC, healthy control; MDD, major depressive disorder; OCD, obsessive compulsive disorder. *False discovery rate corrected *p* < 0.05, a = 0.053.

The characteristics of the subjects were compared using the independent sample *t*‐test, and four multiple comparisons between each patient and control group were corrected using the false discovery rate method.^31^ In the specific condition, subjects with AUD and OCD showed lower use of MB strategies than did matched HCs, and MDD subjects did not differ from HCs (Figure 3 and Table 2). In the flexible condition, AUD subjects exhibited fewer instances of MF strategy use than did matched HC. Additionally, MDD subjects used the MF strategy less frequently than did matched HC subjects in the flexible condition. The arbitration mechanism between MB and MF is characterized by system switching parameters, and our results showed that AUD, OCD and MDD do not differ from matched HCs in specific conditions. However, in flexible conditions, OCD exhibits an increase in switching between learning systems. The results of the correlation analysis showed that both system preferences (specific: r_19_ = −0.09, *p* = 0.71; flexible: r_19_ = −0.12, *p* = 0.64) and system switching (flexible: r_19_ = −0.16, *p* = 0.52) are not correlated with the OCI‐R scores of AUD patients. As expected, there was no difference in system switching between MDD subjects and the matched HC group.

**TABLE 2:** Inferred parameters.

	*N*	System preference		System switching	
		Specific condition	Flexible condition	Specific condition	Flexible condition
AUD	19	50.64(9.56)	44.81(13.04)	47.71(6.03)	52.47(7.70)
HC	19	58.26(9.31)	34.02(10.36)	51.57(6.85)	47.24(7.41)
*t*		2.491*	2.823*	1.842	2.134
FDR corrected *p*		0.034	0.031	0.074	0.053
*d*		0.81	0.92	0.60	0.69
OCD	30	52.84(12.32)	37.01(9.94)	47.77(8.07)	51.46(6.96)
HC	30	61.87(10.03)	32.15(8.83)	50.58(8.33)	46.07(9.91)
*t*		3.115*	2.001	1.329	2.435*
FDR corrected *p*		0.011	0.067	0.189	0.036
*d*		0.80	0.52	0.34	0.63
MDD	20	56.52(9.95)	43.10(10.86)	49.33(7.98)	50.03(6.78)
HC	19	58.58(7.19)	34.12(9.49)	52.77(5.38)	48.44(7.86)
*t*		0.742	2.746*	1.568	0.677
FDR corrected *p*		0.618	0.037	0.251	0.503
*d*		0.24	0.88	0.51	0.22

Abbreviations: AUD, alcohol use disorder; FDR, false discovery rate; HC, healthy control; MDD, major depressive disorder; OCD, obsessive‐compulsive disorder

* FDR corrected *p* < 0.05.

Due to the marginal significant difference in sex between the AUD group and the HC group, we controlled sex as a covariate to conduct ANOVA, and the results showed that the AUD group still preferred the MF strategy in specific trials (F(1, 35) = 8.733, *p* = 0.006) and still preferred the MB strategy (F(1, 35) = 8.042, *p* = 0.008) in flexible trials.

As in the past research,^27^ the difference of preference for learning strategy in different state transition probabilities is not the focus of this study, and the analysis is presented in Figure S2 and Table S2.

Because we found bias in patients' preference for learning systems compared with HC and this preference correlates with subjects' gains (Figure 2), we also analysed patient performance on objective tasks. All patient groups showed lower task performance (defined as the average number of coins obtained per trial) and choice optimality (refers to the percentage of trials in which subjects make the optimal decision sequence [i.e., the decision that leads to the highest possible reward outcome] across all trials) than the HC group, as detailed in Figure S3 and Table S3.

## 4 DISCUSSION

The current study found evidence of impaired arbitration between MB and MF learning in AUD and OCD. Specifically, two aspects of arbitration were impaired. First, AUD and OCD subjects exhibited deficits in selecting the optimal strategy in different task conditions (i.e., showing a preference for suboptimal strategies). In the specific condition, where MB control is optimal, both AUD and OCD subjects showed lower preferences for MB control than did the HC subjects. Second, but more importantly, we measured the switching frequency of subjects between dominant learning systems, which is more representative of the arbitration process. Results showed that in the flexible condition, where MF control is optimal, the switching frequency of only OCD subjects was higher than that of HC subjects, which means that it is difficult for it to maintain stable use of the optimal system. Overall, our findings suggest that disorders of compulsivity are related to the impairment of the arbitration mechanism between MB and MF controls.

Past research on healthy individuals has reported that an increase in environmental complexity can promote the use of MB strategies.^32^ Our study found that disorders of compulsivity disrupted the increased use of MB strategy in the more complex specific trial. A wealth of studies using the two‐step task found that compulsivity impairs goal‐directed control,^5^, ^6^, ^33^, ^34^ perhaps due to their similarity in complexity to the specific conditions used in this paradigm. On the contrary, when the complexity of the environment decreases, maintaining MF control with lower computational costs and reducing switching from MF control to MB control is optimal. We found that in a flexible condition with lower complexity, OCD increased subjects' switching between dominant systems, disrupting the maintenance of MF control use. All of these imply that disorders of compulsivity may be related to the impairment of selection and the maintenance of optimal control.

Previous studies have found impaired representation of RPE in patients with MDD^35^, ^36^ and people with subclinical depression,^37^ as compulsivity often comorbidly accompanies depression.^38^ We found that, in the flexible task condition, where MF control is optimal, AUD and MDD subjects exhibited lower preferences for MF control than did HC subjects; however, neither the use of MB control in the specific condition, where MB control is optimal, nor the maintenance of MF control in flexible conditions, where MF control is optimal, showed significant differences between MDD subjects and matched HC subjects. Based on this, we conclude that damage to the arbitration process is not due to depression.

The finding of impaired arbitration control in disorders of compulsivity further raises the issue of neural mechanisms involved in the impairment. A study conducted with healthy volunteers using the same task found that the estimation of the reliability of the dual system by arbitration control primarily involves the anterior cingulate cortex (ACC) and ventrolateral prefrontal cortex (vlPFC).^10^ The ACC allocates cognitive control over behaviour based on evaluation of the expected value of control^39^ and participates in addressing conflicts between goal‐directed (MB) and habitual (MF) control.^40^ Previous studies found that patients with alcohol dependence exhibit abnormal activation of the ACC in working memory tasks,^41^ heavy drinkers display thinner ACC thickness^42^ and that reduction of the dorsal ACC and dorsomedial PFC grey matter concentration and functional resting‐state connectivity was related to greater alcohol use.^43^ Arbitration control also involves the vlPFC. According to Kim et al.,^44^ the vlPFC is related to a key aspect of arbitration, which is the adjustment of the prediction error baseline. Past research has found that abnormal vlPFC activation increases in alcohol‐dependent subjects under high cognitive working memory load.^45^

This study has several limitations. First, in the current study, there was no significant difference in system switching between AUD patients and matched control after false discovery rate correction, and the OCI‐R scale score was not correlated with system switching and system preference. Therefore, the conclusion of our study is exploratory, and future research can be validated in larger samples. Second, because this is a cross‐sectional study, it is not clear whether these abnormalities are compulsivity‐specific or whether they are due to differences in working memory, IQ and/or other dimensions among patients with disorder of compulsivity and control subjects. Third, all enrolled patients were treated with psychiatric medications, but a recent study found that escitalopram reduced reinforcement sensitivity on the two‐step task.^46^ Final, past research has suggested that an increase in task instruction details can lead to a bias of participants towards using MB strategies,^47^ and the arbitration model was inferior to the MB model in the data of this study (Figure S1), which all imply that caution should be exercised when interpreting the results of sequence learning tasks.

## 5 CONCLUSION

The shared anomaly pattern we reported here suggests that the abnormal arbitration control may be an underlying neurocomputational mechanism, which contributes to the compulsivity dimension common to these disorders. The use of cognitive or pharmacological strategies to shift rigid and single dependence on a certain strategy towards flexible selection and maintenance of the optimal strategy in each context may be useful in treatment.

## AUTHOR CONTRIBUTIONS

*Conceptualization*: Zhongqiang Ruan and Ziwen Peng. *Methodology*: Zhongqiang Ruan and Ziwen Peng. *Formal analysis*: Zhongqiang Ruan. *Investigation*: Shilin Liu and Yu an Liu. *Resources*: Qiong Yang. *Writing—original draft*: Zhongqiang Ruan. *Writing—review and editing*: Ziwen Peng. *Supervision*: Qiong Yang and Ziwen Peng. *Funding acquisition*: Ziwen Peng. All authors contributed to the article and approved the submitted version.

## CONFLICT OF INTEREST STATEMENT

The authors have declared that no competing interests exist.

## ETHICS STATEMENT

This study was approved by the Institutional Research and Ethics Committee of Affiliated Brain Hospital of Guangzhou Medical University and obtained written informed consent from all subjects. All research procedures were in accordance with the ethical standards of national and institutional committees relevant to human experimentation and in accordance with the 1975 Declaration of Helsinki as revised in 2008.

## Supporting information


**Table S1.** Details of pharmaceutical treatments.
**Figure S1.** Model comparison. The arbitration model (Arb) is superior to the model without arbitration (MF alone) in explaining the adaptive decision‐making of subjects. The results of model comparison are consistent on the two indicators of goodness of fit (Akaike information criterion [AIC] and Bayesian information criterion [BIC]). ***p < 0.001.
**Figure S2.** Inferred parameters under high and low uncertainty. Differences in system preferences and system switching between patients with different disorders and matched healthy controls. AUD: Alcohol Use Disorder, OCD: Obsessive Compulsive Disorder, MDD: Major Depressive Disorder. *p < 0.05, **p < 0.01, ***p < 0.001.
**Table S2.** Inferred parameters under high and low uncertainty.
**Figure S3.** Task Performance. AUD: Alcohol Use Disorder, OCD: Obsessive Compulsive Disorder, MDD: Major Depressive Disorder. * FDR corrected *p* < 0.05, ** FDR corrected *p* < 0.01, *** FDR corrected *p* < 0.001.
**Table S3.** Task Performance.

## Data Availability

All code associated with this paper are available online (https://github.com/allhaillelouch0/three-stage-task/ and https://github.com/allhaillelouch0/computational-modeling-code-of-three-stage-task). De‐identified data can be downloaded online (https://github.com/allhaillelouch0/three-stage-task-data/ and https://github.com/allhaillelouch0/subjects-OCD-2023).
