# Supplementary material for: Disorders of compulsivity: Deficits in arbitrating learning strategies
Source: Addict Biol. 2024 Aug 9;29(8):e13433. doi: 10.1111/adb.13433 (PMC11315606; doi:10.1111/adb.13433)
Supplement: Supplementary file 1 — Table S1. Details of pharmaceutical treatments. Figure S1. Model comparison. The arbitration model (Arb) is superior to the model without arbitration (MF alone) in explaining the adaptive decision‐making of subjects. The results of model comparison are consistent on the two indicators of goodness of fit (Akaike information criterion [AIC] and Bayesian information criterion [BIC]). ***p < 0.001. Figure S2. Inferred parameters under high and low uncertainty. Differences in system preferences and system switching between patients with different disorders and matched healthy controls. AUD: Alcohol Use Disorder, OCD: Obsessive Compulsive Disorder, MDD: Major Depressive Disorder. *p < 0.05, **p < 0.01, ***p < 0.001. Table S2. Inferred parameters under high and low uncertainty. Figure S3. Task Performance. AUD: Alcohol Use Disorder, OCD: Obsessive Compulsive Disorder, MDD: Major Depressive Disorder. * FDR corrected p < 0.05, ** FDR corrected p < 0.01, *** FDR corrected p < 0.001. Table S3. Task Performance. [file ADB-29-e13433-s001.docx]

Supplementary Material

**Disorders of compulsivity: Deficits in arbitrating learning strategies**

**Table S1.** Details of pharmaceutical treatments

|  | Treatment | Number of cases | Average dosage (mg) |
| --- | --- | --- | --- |
| AUD (n=19) | escitalopram | 8 | 40 |
|  | escitalopram + sodium valproate | 2 | 40 + 500 |
|  | quetiapine | 5 | 200 |
|  | escitalopram + quetiapine | 3 | 40 + 200 |
|  | paroxetine | 1 | 40 |
|  |  |  |  |
| OCD (n=30) | fluvoxamine | 14 | 200 |
|  | fluvoxamine + quetiapine | 2 | 150 + 200 |
|  | fluvoxamine + tandospirone | 2 | 150 + 40 |
|  | sertraline | 8 | 150 |
|  | sertraline + lithium carbonate | 1 | 150 + 500 |
|  | escitalopram | 2 | 40 |
|  | paroxetine + sodium valproate | 1 | 40 + 500 |
|  |  |  |  |
| MDD (n=20) | sertraline | 8 | 150 |
|  | escitalopram | 5 | 40 |
|  | paroxetine | 4 | 40 |
|  | paroxetine + sodium valproate | 2 | 40 + 825 |
|  | buspirone + duloxetine | 1 | 20 + 50 |

AUD: Alcohol Use Disorder, OCD: Obsessive Compulsive Disorder, MDD: Major Depressive Disorder.


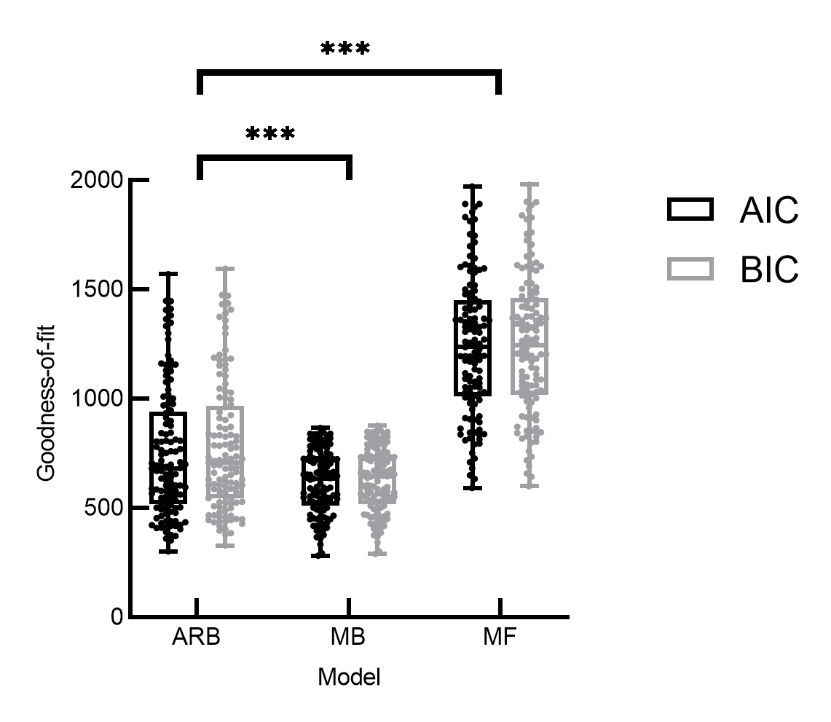


**Figure S1.** Model comparison. The arbitration model (Arb) is superior to the model without arbitration (MF alone) in explaining the adaptive decision-making of subjects. The results of model comparison are consistent on the two indicators of goodness of fit (Akaike information criterion [AIC] and Bayesian information criterion [BIC]). ***p < 0.001.


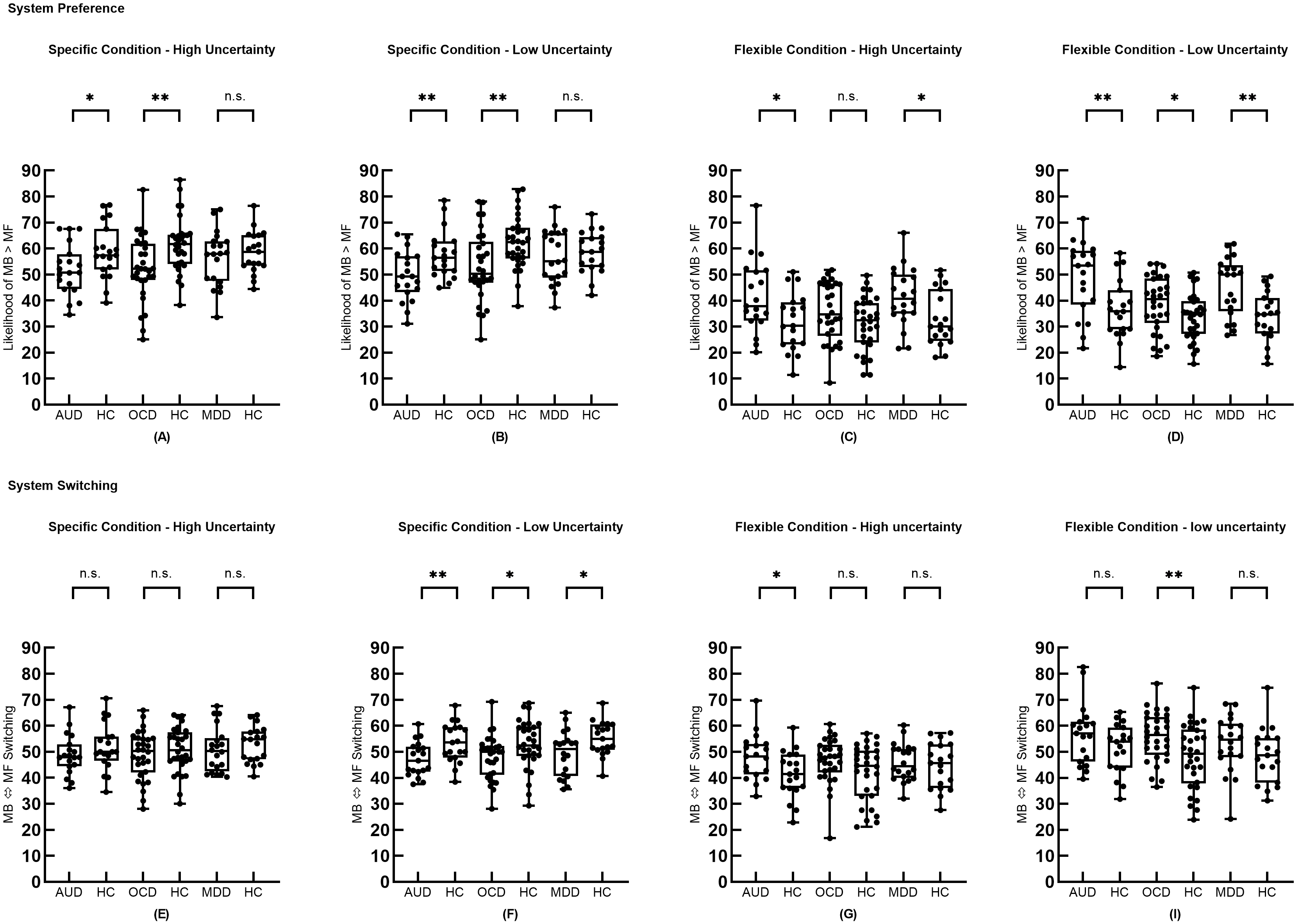


**Figure S2.** Inferred parameters under high and low uncertainty. Differences in system preferences and system switching between patients with different disorders and matched healthy controls. AUD: Alcohol Use Disorder, OCD: Obsessive Compulsive Disorder, MDD: Major Depressive Disorder. *p < 0.05, **p < 0.01, ***p < 0.001.

**Table S2**. Inferred parameters under high and low uncertainty

|  | N | System Preference | | | | System Switching | | | |
| --- | --- | --- | --- | --- | --- | --- | --- | --- | --- |
|  |  | Specific Condition | | Flexible Condition | | Specific Condition | | Flexible Condition | |
|  |  | High Uncertainty | Low Uncertainty | High Uncertainty | Low Uncertainty | High Uncertainty | Low Uncertainty | High Uncertainty | Low Uncertainty |
| AUD | 19 | 51.87  (9.87) | 49.41  (9.63) | 41.74  (14.04) | 48.69  (13.99) | 48.80  (7.72) | 47.17  (6.60) | 47.98  (8.68) | 56.79  (11.73) |
| HC | 19 | 58.84  (10.54) | 57.74  (9.17) | 31.79  (11.05) | 36.72  (11.37) | 51.85  (9.13) | 53.47  (7.60) | 41.27  (9.08) | 51.34  (9.43) |
| t |  | 2.105* | 2.730** | 2.427* | 2.896** | 1.112 | 2.728** | 2.326* | 1.578 |
| *p* |  | 0.042 | 0.010 | 0.020 | 0.006 | 0.274 | 0.010 | 0.026 | 0.124 |
|  |  |  |  |  |  |  |  |  |  |
| OCD | 30 | 52.49  (12.43) | 53.16  (13.32) | 35.75  (11.23) | 38.71  (10.55) | 48.76  (9.24) | 47.84  (8.38) | 46.64  (8.73) | 55.76  (9.48) |
| HC | 30 | 61.49  (10.58) | 62.31  (10.21) | 31.08  (10.33) | 33.56  (9,00) | 50.71  (8.76) | 52.83  (9.49) | 41.83  (10.79) | 48.14  (12.37) |
| t |  | 3.019** | 2.988** | 1.676 | 2.035* | 0.842 | 2.159* | 1.896 | 2.678** |
| *p* |  | 0.004 | 0.004 | 0.099 | 0.046 | 0.403 | 0.035 | 0.063 | 0.010 |
|  |  |  |  |  |  |  |  |  |  |
| MDD | 20 | 56.29  (10.61) | 56.73  (10.36) | 41.32  (11.29) | 45.40  (11.22) | 50.82  (8.61) | 49.26  (8.70) | 46.02  (7.24) | 52.97  (10.90) |
| HC | 19 | 58.48  (8.14) | 58.58  (8.07) | 32.97  (10.52) | 33.91  (9.55) | 52.83  (6.98) | 55.11  (6.56) | 44.74  (8.92) | 48.49  (10.54) |
| t |  | 0.719 | 0.621 | 2.388* | 3.434** | 0.799 | 2.361* | 0.495 | 1.303 |
| *p* |  | 0.477 | 0.539 | 0.022 | 0.001 | 0.429 | 0.024 | 0.624 | 0.201 |

AUD: Alcohol Use Disorder, OCD: Obsessive Compulsive Disorder, MDD: Major Depressive Disorder. *p < 0.05, **p < 0.01, ***p < 0.001.

**
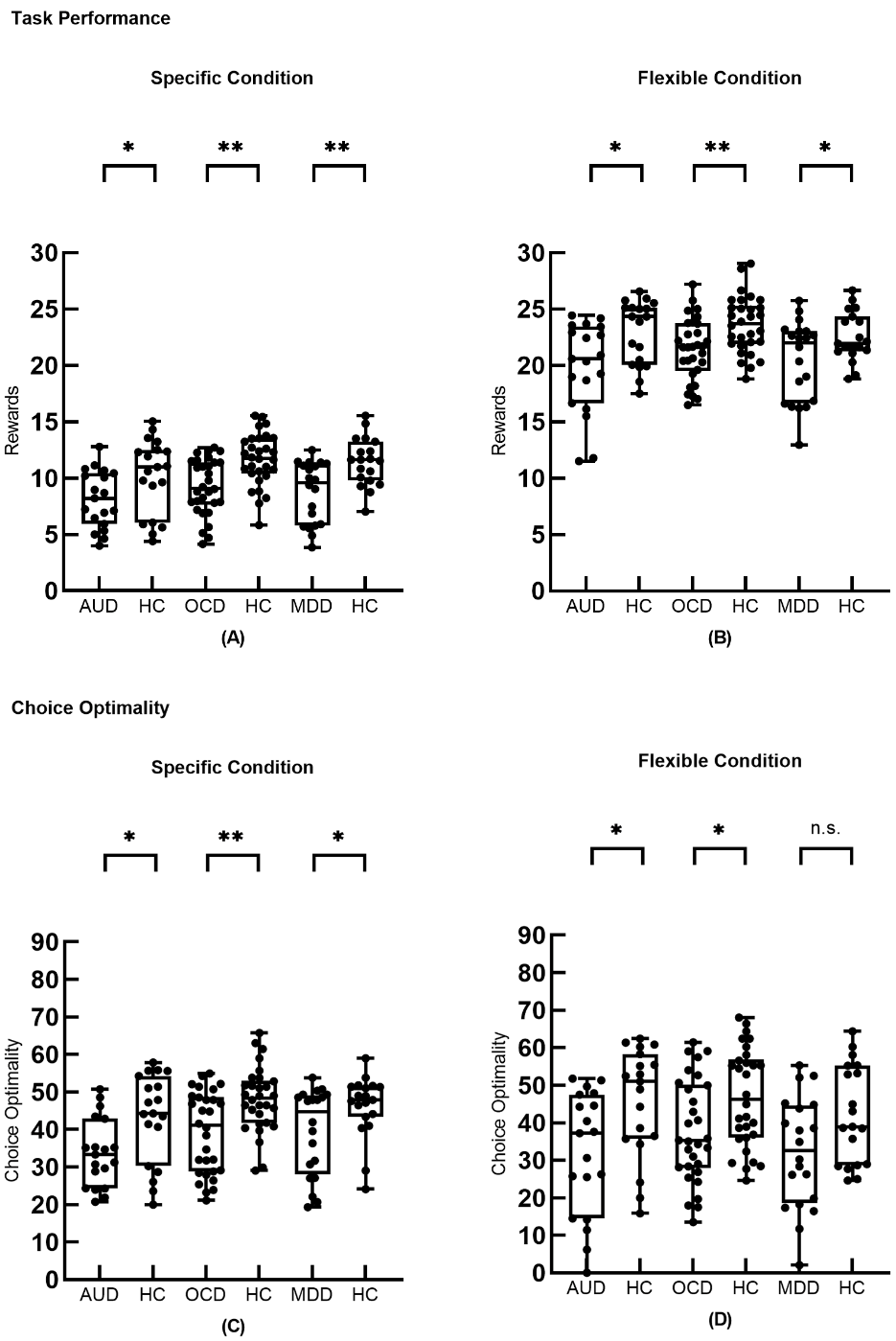
**

**Figure S3**. Task Performance. AUD: Alcohol Use Disorder, OCD: Obsessive Compulsive Disorder, MDD: Major Depressive Disorder. * FDR corrected *p* < 0.05, ** FDR corrected *p* < 0.01, *** FDR corrected *p* < 0.001.

**Table S3**. Task Performance

|  | N | Task Performance | | Choice Optimality | |
| --- | --- | --- | --- | --- | --- |
|  |  | Specific Condition | Flexible Condition | Specific Condition | Flexible Condition |
| AUD | 19 | 8.14 (2.54) | 19.88 (3.99) | 33.74 (9.10) | 31.94 (16.44) |
| HC | 19 | 10.20 (3.31) | 22.98 (2.83) | 43.14 (11.98) | 45.72 (14.59) |
| t |  | 2.149* | 2.757* | 2.722* | 2.734* |
| FDR corrected *p* |  | 0.038 | 0.036 | 0.020 | 0.013 |
|  |  |  |  |  |  |
| OCD | 30 | 9.23 (2.48) | 21.43 (2.74) | 39.26 (10.85) | 37.70 (13.69) |
| HC | 30 | 11.65 (2.32) | 23.62 (2.51) | 48.02 (8.61) | 46.71 (13.16) |
| t |  | 3.897** | 3.222** | 3.462** | 2.598* |
| FDR corrected *p* |  | 0.001 | 0.003 | 0.002 | 0.012 |
|  |  |  |  |  |  |
| MDD | 20 | 8.77 (2.71) | 20.45 (3.56) | 39.53 (11.56) | 32.09 (14.84) |
| HC | 19 | 11.46 (2.14) | 22.54 (2.19) | 46.27 (8.28) | 42.25 (13.12) |
| t |  | 3.428** | 2.222* | 2.102* | 2.262 |
| FDR corrected *p* |  | 0.006 | 0.046 | 0.043 | 0.059 |

AUD: Alcohol Use Disorder, OCD: Obsessive Compulsive Disorder, MDD: Major Depressive Disorder. * FDR corrected *p* < 0.05, ** FDR corrected *p* < 0.01, *** FDR corrected *p* < 0.001.
